# Optimization of a peptide ligand for the adhesion GPCR ADGRG2 provides a potent tool to explore receptor biology

**DOI:** 10.1074/jbc.RA120.014726

**Published:** 2020-12-17

**Authors:** Yujing Sun, Daolai Zhang, Ming-Liang Ma, Hui Lin, Youchen Song, Junyan Wang, Chuanshun Ma, Ke Yu, Wentao An, Shengchao Guo, Dongfang He, Zhao Yang, Peng Xiao, Guige Hou, Xiao Yu, Jin-Peng Sun

**Affiliations:** 1 School of Pharmacy, Binzhou Medical University, Yantai, Shandong, China; 2 Department of Endocrinology, Cheeloo College of Medicine, Qilu Hospital, Shandong University, Jinan, China; 3 Key Laboratory Experimental Teratology of the Ministry of Education and Department of Biochemistry and Molecular Biology, Shandong University School of Medicine, Jinan, Shandong, China; 4 Key Laboratory Experimental Teratology of the Ministry of Education and Department of Physiology and Pathophysiology, School of Basic Medical Sciences, Shandong University, Jinan, Shandong, China; 5 Department of Medical Biophysics, University of Toronto, Toronto, Ontario, Canada; 6 Key Laboratory of Molecular Cardiovascular Science, Department of Physiology and Pathophysiology, School of Basic Medical Sciences, Peking University, Ministry of Education, Beijing, China

**Keywords:** G protein–coupled receptor (GPCR), adhesion G protein–coupled receptor (aGPCR), ADGRG2, *Stachel* peptide agonist, signal transduction, aGPCR, adhesion G protein–coupled receptor, BRET, bioluminescence resonance energy transfer, CTF, C-terminal fragment, ECD, extracellular domain, NTF, N-terminal fragment, TBST, Tris Buffer Saline Tween20

## Abstract

The adhesion GPCR ADGRG2, also known as GPR64, is a critical regulator of male fertility that maintains ion/pH homeostasis and CFTR coupling. The molecular basis of ADGRG2 function is poorly understood, in part because no endogenous ligands for ADGRG2 have been reported, thus limiting the tools available to interrogate ADGRG2 activity. It has been shown that ADGRG2 can be activated by a peptide, termed p15, derived from its own N-terminal region known as the *Stachel* sequence. However, the low affinity of p15 limits its utility for ADGRG2 characterization. In the current study, we used alanine scanning mutagenesis to examine the critical residues responsible for p15-induced ADGRG2 activity. We next designed systematic strategies to optimize the peptide agonist of ADGRG2, using natural and unnatural amino acid substitutions. We obtained an optimized ADGRG2 *Stachel* peptide T1V/F3Phe(4-Me) (VPM-p15) that activated ADGRG2 with significantly improved (>2 orders of magnitude) affinity. We then characterized the residues in ADGRG2 that were important for ADGRG2 activation in response to VPM-p15 engagement, finding that the toggle switch W^6.53^ and residues of the ECL2 region of ADGRG2 are key determinants for VPM-p15 interactions and VPM-p15-induced Gs or arrestin signaling. Our study not only provides a useful tool to investigate the function of ADGRG2 but also offers new insights to guide further optimization of *Stachel* peptides to activate adhesion GPCR members.

The adhesion G protein–coupled receptors (aGPCRs) are a family of 33 receptors in humans that are involved in many diverse biological processes, including brain functions, immune responses, and fertility (1, 2, 3, 4, 5, 6, 7, 8, 9, 10). aGPCRs are unique among all GPCR families with long N termini and multiple domains that are implicated in cell–cell communications and cell–matrix interactions (1, 11, 12). Structurally, aGPCRs consist of a long extracellular domain (ECD), a seven-transmembrane domain (7TM), and an intracellular C-terminal tail (intracellular domain) (13, 14). Another feature of this class of GPCRs is an autoproteolytic site that is present at their GPCR autoproteolysis-inducing (GAIN) domain, which autocleaves aGPCRs into an N-terminal fragment (NTF) and a C-terminal fragment (CTF) for most of their family members (15). In general, the dissociated NTF still binds to the CTF. The GAIN domain and the 7TM bundle of an aGPCR often function as a duet during the activation (13, 16, 17, 18, 19, 20).

There are several proposed activation mechanisms of aGPCRs. One of these mechanisms is the “tethered agonist” model (13, 21). In this model, the GAIN domain autoproteolysis exposes a tethered cryptic agonist sequence, designated as *Stachel* (German for stinger), within the N-terminal region of an aGPCR member, which induces receptor activation through its interaction with the 7TM domain. The tethered *Stachel* sequence located at the CTF plays an important role in aGPCR activation in response to different physiological stimulations, such as ligand binding, mechanostress, or removing of the NTF (13, 18, 19, 20). Furthermore, synthetic peptides mimicking the *Stachel* sequence are called *Stachel* peptides for each aGPCR receptor and have been shown to activate their corresponding aGPCRs (13, 21).

Adhesion G protein–coupled receptor G2(ADGRG2), also known as G protein–coupled receptor 64 (GPR64) or human epididymal gene product 6 (HE6), has attracted substantial attention for its specific expression and essential functions in the male reproductive system (22). Recent studies have found that its deficiency results in the dysfunction of fluid reabsorption and male infertility (5). Like most aGPCRs, ADGRG2 is still regarded as an orphan receptor with no identified endogenous ligands (11). *In vitro*, the overexpression of ADGRG2 caused constitutive Gs- and Gq-coupling activity, and a stronger effect was observed with the CTF truncation mutant ADGRG2 (ADGRG2-β) (5). In our recent studies, we have found that ADGRG2 is specifically coupled to the cystic fibrosis transmembrane conductance regulator (CFTR) through a Gq- and β-arrestin1-mediated mechanism, regulating the ion/water homeostasis in efferent ductules, which is essential for male fertility. It has been shown that ADGRG2 could be activated by the *Stachel* peptide p15 (sequence: TSFGILLDLSRTSLP) derived from the *Stachel* sequence (23), to elicit downstream Gs, Gq, G12/13 signaling (Fig. 1*A*) (23, 24, 25). p15 does not activate or control other aGPCRs (GPR110, GPR133), thereby displaying apparent specificities for the receptor it originated from (23).

**Figure 1:**
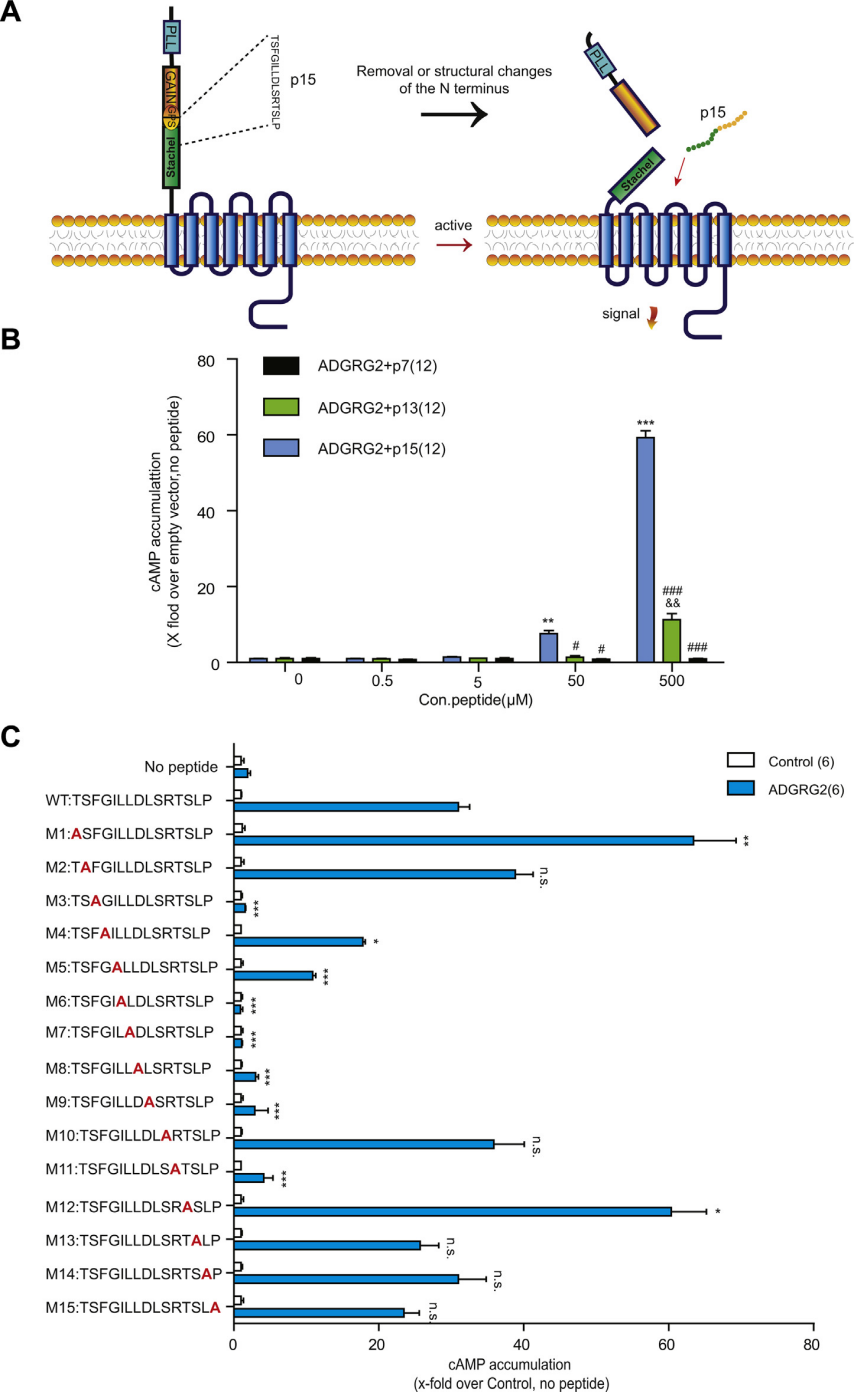
**Critical residues contributed to ADGRG2 tethered peptide p15 binding.***A*, a schematic diagram of the activation of ADGRG2 by autocleavage and the structural rearrangement in the N terminus. Autocatalytic cleavage at the GPS of ADGRG2 results in the removal of theα subunit, followed by the folding of the *Stachel* sequence into the 7TM domain to activate ADGRG2. *B*, activities of truncated ADGRG2 *Stachel* peptides. HEK293 cells were transfected by ADGRG2-full length (ADGRG2-FL) plasmid and stimulated by 0, 0.5, 5, 50, 500 μM ADGRG2 *Stachel* peptide p15, N terminus truncated peptide p13 and both N and C termini truncated peptide p7, respectively. The cAMP levels were monitored by the Glosensor assay. ∗∗*p* < 0.01, ∗∗∗*p* < 0.001, ADGRG2 *Stachel* peptide p15 concentration 50 μM, 500 μM compared with 0 μM. &&*p* < 0.01, ADGRG2 *Stachel* peptide p13 500 μM compared with 0 μM. #*p* < 0.05, ###*p* < 0.001, ADGRG2 *Stachel* peptide p13, p7 compared with p15. Each experiment was repeated 12 times. *C*, a systematic alanine scanning of ADGRG2 *Stachel* peptide p15. HEK293 cells were transfected with ADGRG2-FL, cells transfected with pcDNA3.1 were used as control. Transfected cells were stimulated by 100 μM p15(p15-WT) or its different mutants (p15-Muts), and cAMP levels were detected by the Glosensor assay. n.s. *p* > 0.05, ∗*p* < 0.05, ∗∗ *p* < 0.01, ∗∗∗*p* < 0.001, p15-Muts were compared with p15-WT. Each experiment was repeated six times.

In contrast to other peptidergic GPCRs, *Stachel* sequence–derived synthetic peptides have a significantly low affinity to the 7TM (13, 26). For example, the *Stachel* peptide p15 has a significantly low affinity toward ADGRG2 (23). The low affinity of most *Stachel* peptides limits their *in vivo* studies. Chemical modification of the *Stachel* peptides may help to improve their affinity. Meanwhile, it remains to be revealed how the key residues in the 7TM domain sense the *Stachel* peptide binding and activate the receptor. Gaining more understanding into this question may greatly facilitate the design of peptidomics-based agonists for individual aGPCR members, which remains a great challenge in the field. In the current study, we identified important residues that contributed to the *Stachel* ADGRG2-peptide p15-induced ADGRG2 activation. According to our findings, we designed a systematic strategy to optimize the peptide-derived ADGRG2 agonist. We then obtained an optimized ADGRG2 *Stachel* peptide T1V/F3Phe(4-Me) (VPM-p15) that activated ADGRG2 with significantly improved affinity. Furthermore, we characterized the residues in ADGRG2 that are important for ADGRG2 activation in response to VPM-p15 engagement.

## Results

### The aGPCR ADGRG2 is activated by the *Stachel* peptide p15

First, it is necessary to confirm the activation of *Stachel* peptide p15 on ADGRG2. We generated an ADGRG2 mutant in which the entire ECD (including the entire GPS motif) was removed (ADGRG2-ΔGPS-β). Then, we measured the concentration-dependent p15 activities toward ADGRG2-ΔGPS-β and full-length ADGRG2(ADGRG2-FL) receptor. Increasing the amount of p15 led to a significant increase in cAMP levels, which was determined by the GloSensor assay (Fig. S1*A*). p15 also elicited a rise in the intracellular calcium level, which was determined using a CalfluxVTN Ca^2+^ assay. This change could be inhibited by the Gq/11 selective inhibitor YM-254890, suggesting that p15 induced the calcium influx *via* a Gq-dependent pathway (Fig. S1*B*). Of interest, we found through the bioluminescence resonance energy transfer (BRET) assay that p15 promoted the recruitment of β-arrestin1 and β-arrestin2 to ADGRG2, respectively (Fig. S1, *C*–*D*). It is notable that p15 induced significantly more recruitment of β-arrestin1 or β-arrestin2 through the truncated mutant compared with the wildtype ADGRG2. Moreover, the ADGRG2-β (CTF fragment) shows a significantly higher constitutive activity for arrestin recruitment compared with that of the wildtype ADGRG2 (Fig. S1, *E*–*F*). Because the *Stachel* sequence of ADGRG2-β could have more chance to access to the 7TM domain compared with that of the *Stachel* sequence in the ADGRG2 full length, these results indicate that the arrestin recruitment to ADGRG2 might be strengthened by the activation of ADGRG2 *via Stachel* sequence interaction. Taken together, these data demonstrate that the *Stachel* peptide p15 activates multiple signaling pathways downstream of ADGRG2.

### Alanine scanning within the p15 peptide identified critical amino acids for activity

Among the three *Stachel* peptides of ADGRG2 (7, 13, 15 amino acids in length), p15 induced the strongest ADGRG2 activation (Fig. 1*B*). To find out which amino acid (aa) residues of p15 are required for receptor activation, we performed a systematic alanine scanning. The results showed that mutations of T1A and T12A enhanced p15 activity. Conversely, mutations of any residues from the middle of LLDL at positions ranging from +6 to +9, F3A, or R11A all greatly impaired p15 activity (Fig. 1*C*, Fig. S2 and Table S1).

### Optimization of *Stachel* peptide p15

**Figure 2:**
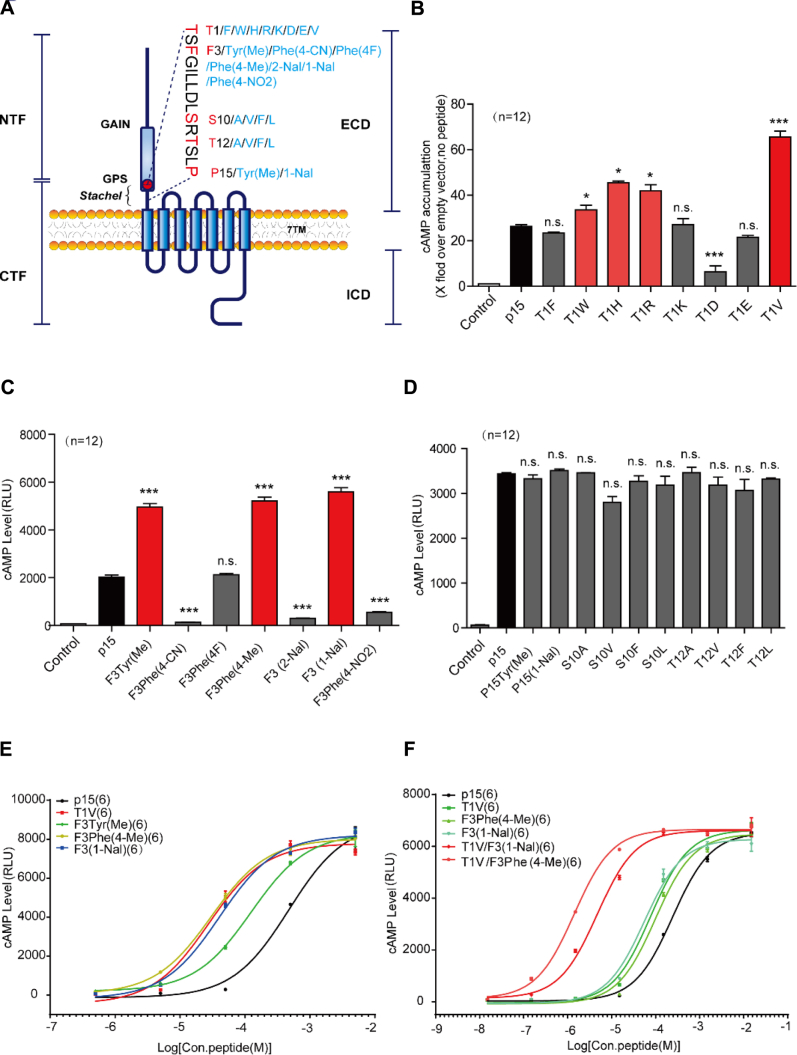
**Optimization of ADGRG2 *Stachel* peptide p15 for higher potency.***A*, a schematic diagram of designed mutants of ADGRG2 *Stachel* peptide p15 according to *Stachel* sequences. *B*–*D*, activities of ADGRG2 *Stachel* peptide p15 mutants at Thr1 (*B*), Phe3 (*C*), Ser10, Thr12, or Pro15 (*D*). HEK293 cells transfected with ADGRG2-ΔGPS-β were stimulated by 100 μM p15 or p15 mutants; PBS solution containing no peptide was used as control vehicle. The cAMP levels were monitored by the Glosensor assay. Each experiment was repeated 12 times. n.s. *p* > 0.05, ∗*p* < 0.05, ∗∗∗*p* < 0.001, signaling activities of ADGRG2 *Stachel* peptide p15 mutants were compared with p15. *E*–*F*, dose–curves of p15, p15 mutants T1V, F3Tyr(Me), F3Phe(4-Me), F3(1-Nal), T1V/F3(1-Nal), and T1V/F3Phe(4-Me). HEK293 cells transfected with ADGRG2-ΔGPS-β were stimulated by increasing concentrations of p15 or p15 mutants. Each experiment was repeated six times.

We next optimized p15 by amino acid substitution, according to the following strategies: (1) diversifying the noncritical residues identified in the alanine scanning to amino acids with different properties; (2) changing critical amino acids into natural or unnatural amino acids with different structures but similar properties (hydrophobicity, polarity, or charge); (3) enhancing the hydrophobicity of the C terminus of the *Stachel* peptide (Fig. 2*A*).

First, we converted the Thr(T) at +1 position to F, W, H, R, K, D, E, V, respectively. Following strategy 1, the substitution of the polar T to a hydrophobic V at +1 position in T1V peptide induced about three times more cAMP accumulation compared with the wildtype p15, as determined by the GloSensor assay (Fig. 2*B*). Next, we changed the Ser(S) at +10 position to A, V, F, L; Thr(T) at +12 position to A, V, F, L to diversify the residue properties at these positions following strategy 2. We also changed +15 position to Tyr(Me) or P15(1-Nal), following strategy 3. However, these p15 mutants did not exhibit enhanced signal transduction capacities compared with the wildtype p15 (Fig. 2*D*). Finally, according to strategy 2, we mutated the Phe(F) at +3 position to unnatural amino acids Tyr(Me), Phe(4-CN), Phe(4F), Phe(4-Me), F3(2-Nal), F3(1-Nal), Phe(4-NO2). Interestingly, the F3Tyr(Me), F3Phe(4-Me), and F3(1-Nal) mutants showed higher signal transduction activities (Fig. 2*C*). According to the results of the above studies, concentration–response curves with p15 mutants T1V, F3Tyr(Me), F3Phe(4-Me), and F3(1-Nal) showed a lower EC_50_ value than wildtype p15 in the same second messenger assay (Fig. 2*E*). To further enhance the potency of the p15 mutants, we performed combined mutations of p15 for those who showed improvement at different p15 positions. The double mutants T1V/F3(1-Nal) and T1V/F3Phe(4-Me) (VPM-p15) showed a significant lower EC_50_ than other mutants, and the effect of the mutant VPM-p15 on EC_50_ was most profound, with an EC_50_ of 1.41 ± 0.16 μM and an approximately 170-fold increase compared with the wildtype p15 peptide (Fig. 2*F* and Table S2). In summary, we obtained an optimized ADGRG2 *Stachel* peptide VPM-p15 that more potently activated ADGRG2 compared with the wildtype p15 peptide.

### VPM-p15 enhances the recruitment of β-arrestin by ADGRG2

**Figure 3:**
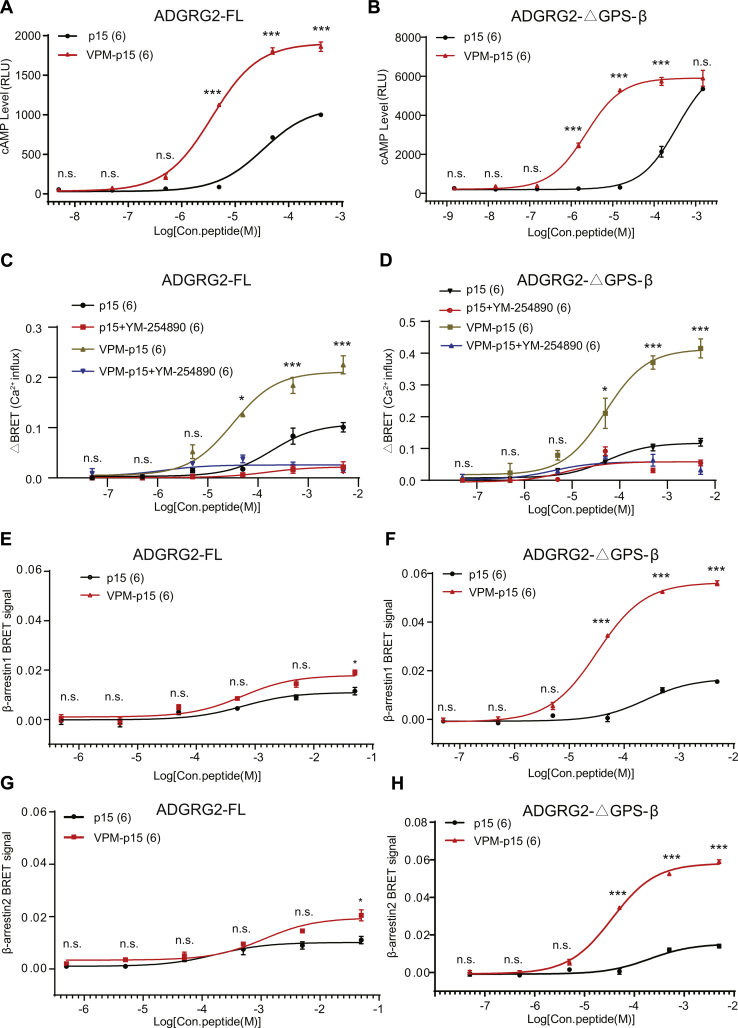
**Signaling properties of ADGRG2 activated by optimized ADGRG2 *Stachel* peptide p15 T1V/F3Phe(4-Me) (VPM-p15).***A*–*H*, signaling properties of ADGRG2-FL or ADGRG2-ΔGPS-β activated by optimized ADGRG2 *Stachel* peptide VPM-p15. HEK293 cells transfected with ADGRG2-FL (*A*, *C*, *E*, *G*) or ADGRG2-ΔGPS-β (*B*, *D*, *F*, *H*) were stimulated by increasing the concentration of ADGRG2 *Stachel* peptide p15 or VPM-p15. cAMP levels were detected by the Glosensor assay (*A*–*B*), Ca^2+^ signaling activities were detected by the CalfluxVTN Ca^2+^ assay (*C*–*D*), β-arrestin1 or β-arrestin2 recruitment was detected by BRET (*E*–*H*). n.s. *p* > 0.05, ∗*p* < 0.05, ∗∗∗*p* < 0.001, signaling activities of VPM-p15 were compared with p15. *A*–*H*, each experiment was repeated six times.

ADGRG2 has been shown to maintain the fluid reabsorption through coupling to both Gq and β-arrestin1 (5). We therefore tested the abilities of VPM-p15 on ADGRG2 to activate Gq by CalfluxVTN Ca^2+^ assay and to recruit β-arrestin by BRET assay. Concentration–response curves showed that VPM-p15 enhanced the abilities of ADGRG2 to activate both Gs and Gq (the Gq/11 selective inhibitor YM-254890 could reduce the intracellular calcium level), in both ADGRG2-FL, ADGRG2-ΔGPS-β overexpressed HEK293 cells (Fig. 3, *A*–*D*). Subsequently, VPM-p15 promoted the recruitment of β-arrestin1 or β-arrestin2 to the ADGRG2 truncated mutant ADGRG2-ΔGPS-β, ADGRG2-β, and ADGRG2-FL (Fig. 3, *E*–*H*, Fig. S3, *A*–*B*). Therefore, the optimized VPM-p15 induced both G protein and arrestin activations downstream of ADGRG2.

### Expression of ADGRG2 and its mutants on the cell surface

**Figure 4:**
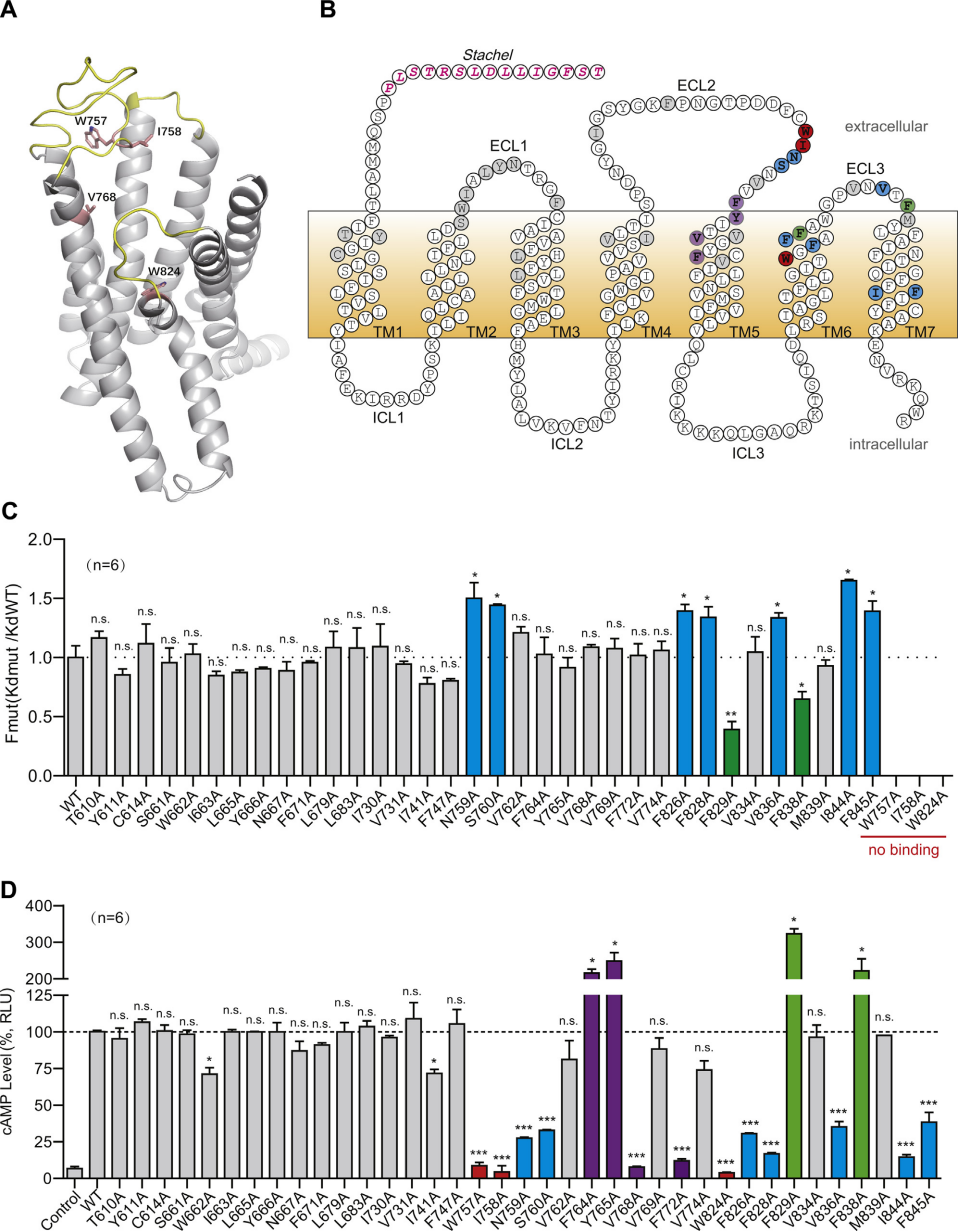
**Key residues of ADGRG2 for VPM-p15 binding and signal transduction.***A*, a cartoon presentation of ADGRG2-ΔGPS-β highlighting the existence of the possible interactions between the VPM-p15 ligand and the binding site. The ADGRG2 structure was modeled by using PTH1R (Protein Data Bank: 6NBI) as a template. The extracellular loops are colored *yellow* and the ligand-binding residues are shown as side chain types and colored *pink*. *B*, a schematic serpentine representation of the ADGRG2 7TM domain residues highlighting its mutation sites. Extracellular and intracellular loops (ECL and ICL) are indicated (*B*). *C*, binding capacities of ADGRG2-ΔGPS-β WT or its mutants for VPM-p15 monitored by BRET experiments. HEK293 cells were transfected with Rluc-ADGRG2-ΔGPS-β or mutants. Transfected cells were stimulated by increasing the concentration of FITC-VPM-p15. Binding capacities were determined by BRET. *Kd* values of ADGRG2-ΔGPS-β WT and its mutants for binding VPM-p15 were calculated by GraphPad. n.s. *p* > 0.05, ∗*p* < 0.05, ∗∗*p* < 0.01, Binding capacities of ADGRG2-ΔGPS-β mutants were compared with ADGRG2-ΔGPS-β WT. *D*, effects of ADGRG2-ΔGPS-β mutants on VPM-p15-induced cAMP accumulation. HEK293 cells transfected with ADGRG2-ΔGPS-β or its mutants were stimulated by 100 μM VPM-p15. cAMP levels were detected by the Glosensor assay. Data were normalized by paralleling experiments with ADGRG2-ΔGPS-β WT. n.s. *p* > 0.05; ∗*p* < 0.05; ∗∗∗*p* < 0.001; ADGRG2-ΔGPS-β mutants were compared with ADGRG2-ΔGPS-β WT. *C*–*D*, each experiment was repeated six times.

To provide a foundation for further optimization of the ligand that activates ADGRG2, we then investigated the molecular mechanism of the engagement of VPM-p15 with ADGRG2. Through sequence alignment, we found that ADGRG2 *Stachel* sequences of the region p15 are highly conserved among different species (Fig. S4*A*). Because adhesion GPCRs are most homologous to class B GPCRs, we therefore used the PTH1R (a class B GPCR) and a Swiss-model software to generate an ADGRG2 three-dimensional coordinate model (Fig. 4*A*). According to this model, we then selected ADGRG2 mutations in extracellular loops 1, 2, 3, and transmembrane helices and then mutated them to alanine by QuikChange (Fig. 4, *A*–*B* and Fig. S4*A*). We transfected HEK293 cells with ADGRG2-ΔGPS-β WT or its corresponding mutants and detected the expression of receptors on the cell surface by cell-surface ELISA (Fig. S4*B*). We adjusted the amount of transfected plasmid and used the mutants without significant alterations of receptor-surface expression for further delineation of the ADGRG2-VPM-p15 engagement.

### Identification of important ADGRG2 residues mediating VPM-p15 engagement

The binding capacities of ADGRG2 WT or its mutants for VPM-p15 were determined by BRET assay, which has been used in previous studies (27, 28, 29). We fused the N terminus of ADGRG2 with a Rluc fragment and coupled FITC to the C terminus of VPM-p15 to facilitate BRET measurement. We stimulated the HEK293 cells transfected with Rluc-ADGRG2-ΔGPS-β wildtype (WT) or mutants by increasing the concentration of FITC-conjugated VPM-p15, and their binding abilities were determined by fitting the data with the saturation curves.

The concentration–response curves of the binding abilities of the receptors to VPM-p15 are shown in the supplemental materials (Fig. S5 and Table S3). Of importance, mutations of two residues in ECL2, including W757^ECL2^ and I758^ECL2^, as well as the mutations of W824^6.53^ in TM6 (superscripts are based on Wooten’s numbering system for class B family GPCRs (30)), almost abolished the binding of VPM-p15 to ADGRG2 (Fig. 4*C* and Table S3). The binding results indicate that the TM6 and ECL2 regions are important for mediating the binding of VPM-p15 to ADGRG2.

We then measured the effects of ADGRG2 mutants on VPM-p15-elicited cAMP accumulation. Among the 37 screened mutants, 12 significantly decreased their VPM-p15-induced ADGRG2 activities (Fig. 4*D* and Table S4). Three (W757^ECL2^, I758^ECL2^, and W824^6.53^) abolished intracellular cAMP accumulation, whereas F829 and F838 enhanced cAMP accumulation, and N759 ^ECL2^, S760 ^ECL2^, F826^6.55^, F828^6.57^, V836^ECL3^, I844^7.53^, and F845^7.54^ might reduce cAMP accumulation. The other residues, including F764^5.35^, Y765^5.36,^ V768^5.39^, and F772^5.43^ may regulate receptor dynamics, which are important for transducing the ligand–binding signal to downstream coupled G proteins (Fig. 4*D* and Table S4).

Both p15 and VPM-p15 were able to induce β-arrestin recruitment *via* ADGRG2. We therefore randomly picked several mutants that did not affect ligand binding and examined their effects on arrestin recruitment. Interestingly, two mutations, F764A and Y765A, showed improved activities toward both G protein and arrestin. In contrast, the mutations of V768A and F772A impaired both G protein and arrestin activities (Fig. 4*D* and Fig. S6, *A*–*B*). These results indicate that G protein and arrestin activity are mediated by at least several common residues located in the ADGRG2 7TM region.

## Discussion

ADGRG2 belongs to the aGPCR subfamily, and many members of this family have been shown to function through G protein coupling (11, 12). Our previous studies have shown that ADGRG2 constitutively activates Gs and Gq, which is consistent with several parallel studies assessing artificial ligands in specific cellular contexts (1, 23). In addition to constitutive activities, tethered agonist sequences have been identified for several aGPCRs, namely, ADGRG6(GPR126), ADGRD1(GPR133) (13), ADGRF1(GPR110), ADGRG1(GPR56) (21), ADGRG2 (GPR64) (23), ADGRG5(GPR114) (31), and the ADGRL(latrophilin) subfamily homolog LAT-1 (32), which function to activate G protein signaling. The length of the most efficient *Stachel* peptides varies between 7 and 18 amino acids (pADGRG6, 16 aa (13); pADGRD1, 13 aa (13); pADGRF1, 12 aa (21); pADGRG1, 7 aa (21); pADGRG2, 15 aa (23); pADGRG5, 18 aa (31); pLPHN-1, 12 aa (32)). Similar to these studies, our data indicated that p15, derived from the ADGRG2 *Stachel* sequence, promoted G protein signaling downstream of ADGRG2 (23, 24, 25). In parallel with G protein signaling, arrestins mediate important functions downstream of many GPCRs (33, 34, 35, 36). In this study, we found that the ADGRG2-β (CTF fragment) showed a higher constitutive activity for arrestin recruitment compared with that of the full-length ADGRG2 (Fig. S1, *E*–*F*). Consistently, a recent study reported that ΔNTF and P622 mutants of ADGRG2 interacted with β-arrestin1 and β-arrestin2, which induced constitutive internalization of ADGRG2 in steady states (24). Therefore, the arrestin recruitment to ADGRG2 might be strengthened by activation of the ADGRG2 *via Stachel* sequence interaction. Moreover, we found that p15 promoted the recruitment of β-arrestin1 or β-arrestin2 to ADGRG2, although the required concentration of p15 was relatively high. By applying our newly developed agonist VPM-p15, we further demonstrated that the arrestins could be recruited to ADGRG2 in an agonist-dependent manner. The small differences between previous results and ours regarding the p15-induced arrestin recruitment to wildtype ADGRG2 may be due to altered experimental methods with different sensitivities (24).

**Figure 5:**
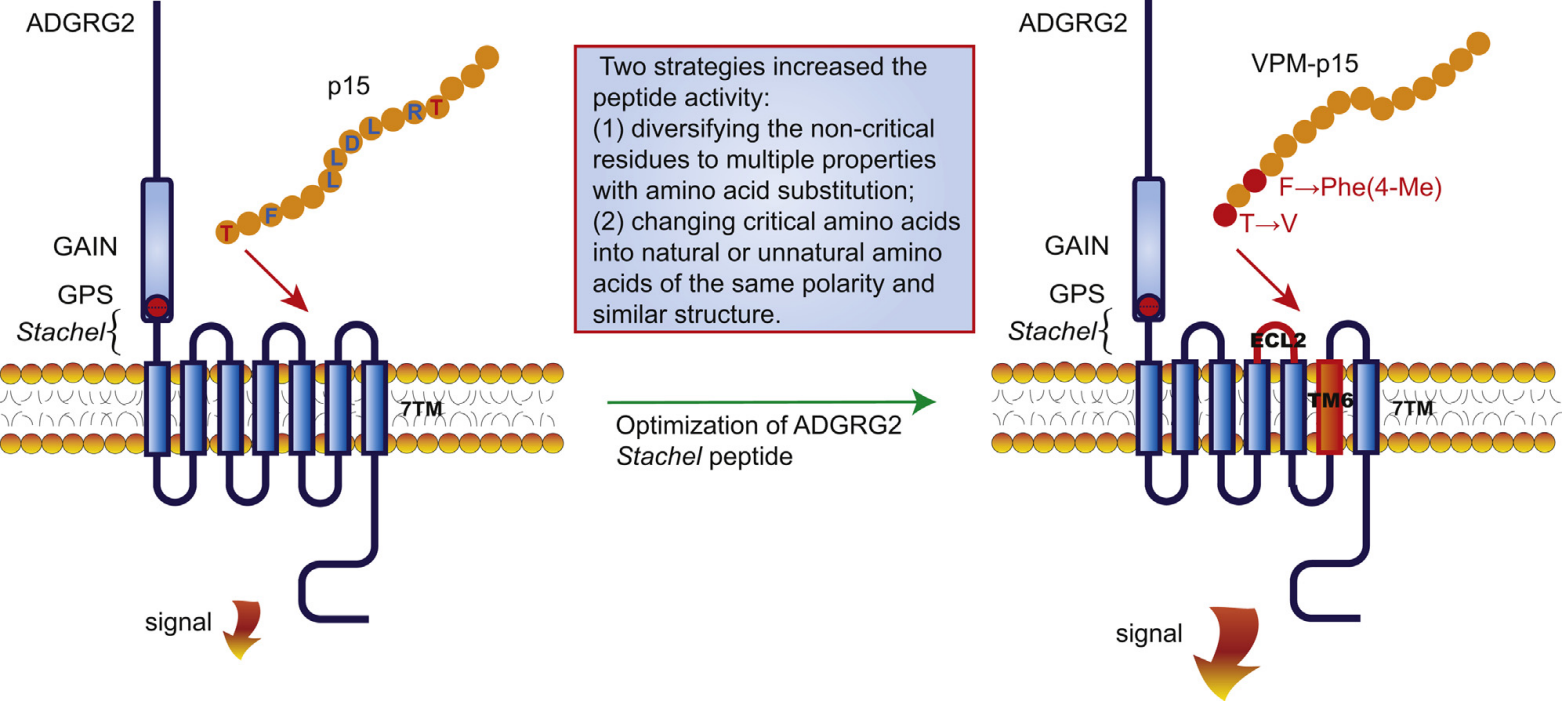
**Schematic diagram depicting the optimization strategies of *Stachel* peptides and the activation of ADGRG2.** ADGRG2 could be activated by the *Stachel* peptide p15 derived from the *Stachel* sequence. However, the *Stachel* peptide p15 has a significantly low affinity toward ADGRG2. We then designed systematic strategies to optimize the peptide agonist of ADGRG2, using natural or unnatural amino acid substitutions. Subsequently, we obtained an optimized ADGRG2 *Stachel* peptide VPM-p15 that activates ADGRG2 more potently compared with the wildtype p15 peptide. Then, the binding results indicate that TM6 and ECL2 regions are important positions to constitute ADGRG2 ligand binding, which recognizes VPM-p15.

The obstacle in working with *Stachel* peptides is that they require large amounts to elicit significant activation levels *in vitro* with EC_50_ values ranging from ∼80 to 400 μM in general (26). The reason for this low-affinity interaction can be explained by the physiologic 1:1 stoichiometry of the *Stachel* agonist and the 7TM interface, which makes high-affinity agonists dispensable. At present, the mechanism underlying the interaction between aGPCR and *Stachel* ligands remains to be solved, and a systematic way to acquire a high-affinity *Stachel* ligand for aGPCR member is in great need to facilitate further aGPCR studies. Here, by performing the alanine scanning of p15, which has been known to activate ADGRG2, we identified the key residues, including F^3^, L^6^LDL^9^, and R^11^, that are important for p15-induced ADGRG2 activity. According to this achievement, we then performed systematic modifications to improve peptide affinity. Among the three strategies that we have exploited, we found two that significantly increased peptide activity. These strategies include (1) diversifying noncritical residues to natural or unnatural amino acids with different properties and (2) changing critical amino acids into natural or unnatural amino acids with similar properties with different structures (Fig. 5). These findings may provide valuable guidance for future peptide agonists optimization toward other aGPCR members.

Currently, no atomic-resolution structures of any aGPCRs have been determined, which brings great challenges to agonist development toward individual aGPCR members. We therefore performed alanine scanning of a group of residues within the upper part of the 7TM domain of ADGRG2 to examine its engagement with VPM-p15. The results suggest that the extracellular loop 2 and the conserved toggle switch W^6.53^ are essential for VPM-p15 binding (Figs. 4 and 5). These clues may also help further peptide agonist design for ADGRG2.

Future drug development for these aGPCRs will benefit greatly from further understanding of the mechanism of aGPCR activation in response to *Stachel* peptides, by using structural, biochemistry, and biophysics methods. Considering the number of human diseases associated with aGPCR mutations and interesting phenotypes observed after aGPCR gene deletion (1, 37), there are compelling reasons to argue that the optimization of *Stachel* peptides for aGPCR activation will contribute to the development of new therapies for human diseases.

## Experimental procedures

### Materials

The GloSensor cAMP Assay (E1290) and Dual-Luciferase Reporter Assay System (E1960) were purchased from Promega. Tetramethylbenzidine was purchased from Thermo Fisher. Coelenterazine 400a (FP-BB839B) was purchased from Interchim. Gq/11 selective inhibitor YM-254890 (AG-CN2-0509-M001) was purchased from Adipogen. All other reagents or chemicals were purchased from Sigma-Aldrich unless otherwise specified.

### Constructs

Wildtype ADGRG2 full-length (ADGRG2-FL) was cloned from mouse total cDNA libraries using the following primers: forward, ATTCTCGAGGATGCTTTTCTCTGGTGGG; reverse, ATTGAATTCCATTTGCTCGATAAAGTG, and then ADGRG2-FL, ADGRG2 C-terminal truncation mutant (ADGRG2-β), and ADGRG2-ΔGPS-β (the entire ECD of ADGRG2 was deleted) were subcloned into the pcDNA3.1 expression vector. The ADGRG2-ΔGPS-β mutants (T610A, Y611A, C614A, S661A, W662A, I663A, L665A, Y666A，N667A, F671A, L679A, L683A, I730A, V731A, F741A, F747A, W757A, I758A, N759A, S760A, V762A, F764A, Y765A, V768A, V769A, F772A, V774A, W824A, F826A, F828A, F829A, V834A, V836A, F838A, M839A, I844A, F845A) were generated using a QuikChange Mutagenesis Kit (Stratagene). All mutations were verified by DNA sequencing. All primers are listed in Table S5.

### Peptide synthesis and solubilization

ADGRG2 *Stachel* peptides were synthesized using standard Fmoc-chemistry on an automated peptide synthesizer MultiPep (Intavis AG). Final side chain deprotection and cleavage from the solid support was achieved by using TFA, water, and thioanisole (95:2.5:2.5vol%). Peptides were subsequently purified to >95% purity by preparative RP-HPLC (Shimadzu LC-8). Peptides were suspended in dimethyl sulfoxide and diluted to ≤4% dimethyl sulfoxide (vol/vol) in experiments.

### Cell culture and transfection

Human embryonic kidney 293 (HEK293) cells were maintained in Dulbecco’s modified Eagle’s medium supplemented with 10% fetal bovine serum, penicillin (100 IU/ml), and streptomycin (100 μg/ml) as described (6, 38). For receptor or other protein expression, plasmids carrying the desired genes were transfected into cells using Lipofectamine 2000 (Invitrogen).

### GloSensor cAMP assay

The Glosensor cAMP assay was performed as described (6, 39, 40). HEK293 cells were transfected with the GloSensor plasmid and the desired expression plasmids in 24-well dishes. Twenty-four hours after transfection, the cells were plated on 96-well plates at a cell density of 20,000 cells/well. The cells were maintained in Dulbecco’s modified Eagle’s medium for another 24 h, washed with PBS, and then incubated with 100 μl of solution containing 10% fetal bovine serum, 2% (v/v) GloSensor cAMP reagent, and 88% CO_2_-independent medium in each well for 2 h. The cAMP signal was examined using a luminescence counter (Mithras LB 940).

### CalfluxVTN Ca^2+^ assay

For the Ca^2+^ assays, HEK293 cells in 24-well dishes were cotransfected with plasmids encoding ADGRG2 or its mutants, CalfluxVTN constructs were used at a 6:1 ratio. Empty vector pcDNA3.1(+) was used to normalize the total amount of transfected plasmid DNA. The transfection and CalfluxVTN Ca^2+^ assays were performed as previously reported (28, 29, 41) with minor modifications. In brief, 24 h post transfection, cells were harvested and distributed in 96-well flat-bottomed white microplates. After another 24 h, the cells were processed accordingly and then were added with the Nluc substrate furimazine (5 μM). The BRET signal was calculated by measuring the ratio of the light emitted by the Venus reporter (535 nm) relative to light emitted by the Nluc reporter (475 nm). The average baseline value (basal BRET ratio) recorded prior to agonist stimulation was subtracted from the experimental BRET signal values to obtain the ΔBRET ratio.

### Bioluminescence resonance energy transfer assay

The ADGRG2-YFP and β-arrestin1-Rluc or β-arrestin2-Rluc plasmids were transiently transfected into HEK293 cells. Forty-eight hours after transfection, the cells were washed with PBS, detached with PBS and 5 mM EDTA, and resuspended in PBS with 0.1% (w/v) glucose at room temperature. The cells were then distributed (80 μg of protein per well) in a 96-well microplate (Corning Inc, Corning, NY, USA) and incubated in the presence or absence of ADGRG2 *Stachel* peptides for 1 min. BRET between Rluc and YFP was measured after the addition of the Rluc substrate coelenterazine 400a (5 μM, Interchim) under a Thermo plate reader. The BRET signal was calculated as the ratio of emission of YFP (527 nm) to Rluc (370–480 nm).

In the detection of binding capacities of ADGRG2 WT or its mutants for VPM-p15, HEK293 cells were transfected with Rluc-ADGRG2-ΔGPS-β or mutants. Transfected cells were stimulated by increasing the concentration of FITC-conjugated VPM-p15. Binding capacities were determined by BRET, EC_50_ values of ADGRG2 WT and its mutants for binding VPM-p15 were calculated by GraphPad, and data were normalized by EC_50_ of WT.

### Cell-surface ELISA assay

After transfection for 24 h, HEK293 cells were cultured at 37 °C in 24-well plates pretreated with polylysine. After another 24-h incubation, the cells were washed with Tris Buffer Saline Tween20 (TBST) once and fixed with 4% paraformaldehyde at room temperature for 10 min. Then the cells were washed three times with TBST and sealed with 5% bovine serum albumin solution for 1 h at room temperature. After three washes with TBST, the cells were incubated with primary antibody overnight at 4 °C. After another three washes with TBST, secondary antibody was added, and the cells were incubated for 1 h at room temperature. Tetramethylbenzidine was added after washing three times with TBST. After 5 to 20 min, the reaction was stopped with 0.25 M HCl solution until the color of the solution changed appropriately to blue. Then the reaction solution was transferred to a 96-well plate, and the absorbance at OD450 was measured with a microplate reader.

### Statistics

All the data are presented as the mean ± SEM from at least three independent experiments. Statistical comparisons were performed using an ANOVA with GraphPad Prism 6.0 (GraphPad Software, San Diego, CA, USA). Significant differences were accepted at *p* < 0.05. The sequence alignments were performed using T-coffee.

## Data availability

The structure presented in this paper has been deposited in the Protein Data Bank with the following code: 6NBI. All remaining data are contained within the article.

## Conflict of interest

The authors declare no conflicts of interest in regards to this manuscript.
