# Supplementary material for: Optimization of a peptide ligand for the adhesion GPCR ADGRG2 provides a potent tool to explore receptor biology
Source: J Biol Chem. 2020 Dec 17;296:100174. doi: 10.1074/jbc.RA120.014726 (PMC7948503; doi:10.1074/jbc.RA120.014726)
Supplement: Supplementary Figures and Tables [file mmc1.docx]

Supporting Information

**Optimization of a peptide ligand for the adhesion GPCR ADGRG2 provides a potent tool to explore receptor biology**

Yujing Sun^2,3,4#^, Daolai Zhang^1,3,4#^ Ming-Liang Ma^3,4#^, Hui Lin^3,4^, Youchen Song^5^, Junyan Wang^3,4^, Chuanshun Ma^1,3,4^, Ke Yu^1^, Wentao An^3,4^, Shengchao Guo^3,4^, Dongfang He^3,4^, Zhao Yang^3,4^, Peng Xiao^3,4^, Guige Hou^1^, Xiao Yu^4^*, Jin-Peng Sun^1,3,6^*

^1^School of Pharmacy, Binzhou Medical University, Yantai, Shandong, 264003, China.

^2^Department of Endocrinology, Qilu Hospital, Cheeloo college of medicine, Shandong University, Jinan, 250012, China.

^3^Key Laboratory Experimental Teratology of the Ministry of Education and Department of Biochemistry and Molecular Biology, Shandong University School of Medicine, 44 Wenhua Xi Road, Jinan, Shandong, 250012, China.

^4^Key Laboratory Experimental Teratology of the Ministry of Education and Department of Physiology and Pathophysiology, School of Basic Medical Sciences, Shandong University, 250012 Jinan, Shandong, China.

^5^Department of Medical Biophysics, University of Toronto, Toronto, ON, M5S 1A8, Canada.

^6^Department of Physiology and Pathophysiology, School of Basic Medical Sciences, Peking University, Key Laboratory of Molecular Cardiovascular Science, Ministry of Education, 100191 Beijing, China.

# These authors contributed equally.

* Corresponding authors:

Jin-Peng Sun (corresponding author) Email: [sunjinpeng@sdu.edu.cn](mailto:sunjinpeng@sdu.edu.cn)

Xiao Yu (corresponding author) Email: [yuxiao@sdu.edu.cn](mailto:yuxiao@sdu.edu.cn)

**Running title:** Optimization of ADGRG2 peptide agonist

**Keywords:** G protein-coupled receptor (GPCR); Adhesion G protein-coupled receptor (aGPCR); ADGRG2; *Stachel* peptide agonist; signal transduction


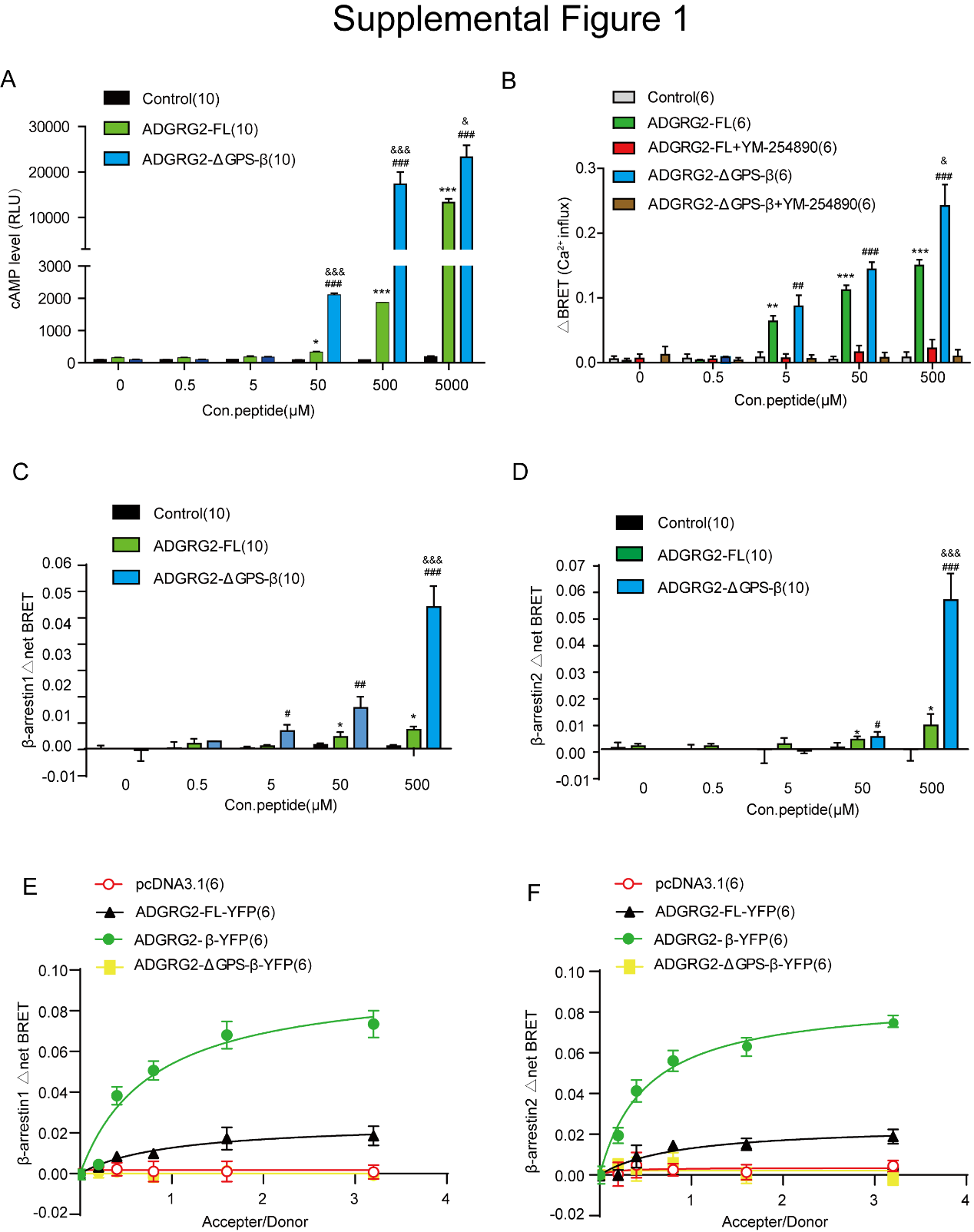


**Supplemental Figure 1. Signaling properties of ADGRG2 activated by ADGRG2 *Stachel* peptide p15.**

**A-D.** Signaling profiles of the full-length ADGRG2 (ADGRG2-FL) or truncated ADGRG2 β subunit by deleting *Stachel* sequence (ADGRG2-△GPS-β) in response to the ADGRG2 *Stachel* peptide p15 derived from ADGRG2 *Stachel* sequence. HEK293 cells were transfected with ADGRG2-FL and ADGRG2-△GPS-β, cells transfected with pcDNA3.1 were used as control. (A) Transfected cells were stimulated by increasing the concentration of ADGRG2 *Stachel* peptide p15; cAMP levels were detected by the Glosensor assay; (B) Ca^2+^ signaling activities were examined by the CalfluxVTN Ca^2+^ assay; (C-D) β-arrestin1 or β-arrestin2 recruitment was detected by BRET (Bioluminescence Resonance Energy Trans). Note: * *P*<0.05, ** *P*<0.01, ****P*<0.001, HEK293 cells transfected with ADGRG2-FL were stimulated by different ADGRG2 *Stachel* peptide p15 concentrations were compared with 0 μM. #*P*<0.01, ## *P*<0.05, ###*P*<0.001, HEK293 cells transfected with ADGRG2-△GPS-β were stimulated by different ADGRG2 *Stachel* peptide p15 concentrations and were compared with 0 μM. &, *P<*0.05, &&, *P*<0.01, &&&, *P<*0.001, ADGRG2-△GPS-β signaling activities were compared with ADGRG2-FL signaling activity. Each experiment was repeated for at least 6 times. **E-F.** The constitutive recruitment of β-arrestin1(E) or β-arrestin2(F) by ADGRG2-FL, ADGRG2-β (ADGRG2-GPS-truncation) or ADGRG2-△GPS-β were detected by BRET assay. HEK293 cells were co-transfected with stable β-arrestin1 or β-arrestin2 concentration (1μg, as the Donor) and increasing concentrations of ADGRG2-FL, ADGRG2-β or ADGRG2-△GPS-β (0 μg, 0.2 μg, 0.4 μg, 0.8 μg, 1.6 μg, 3.2 μg, as the Accepter) then the BRET signals were detected.


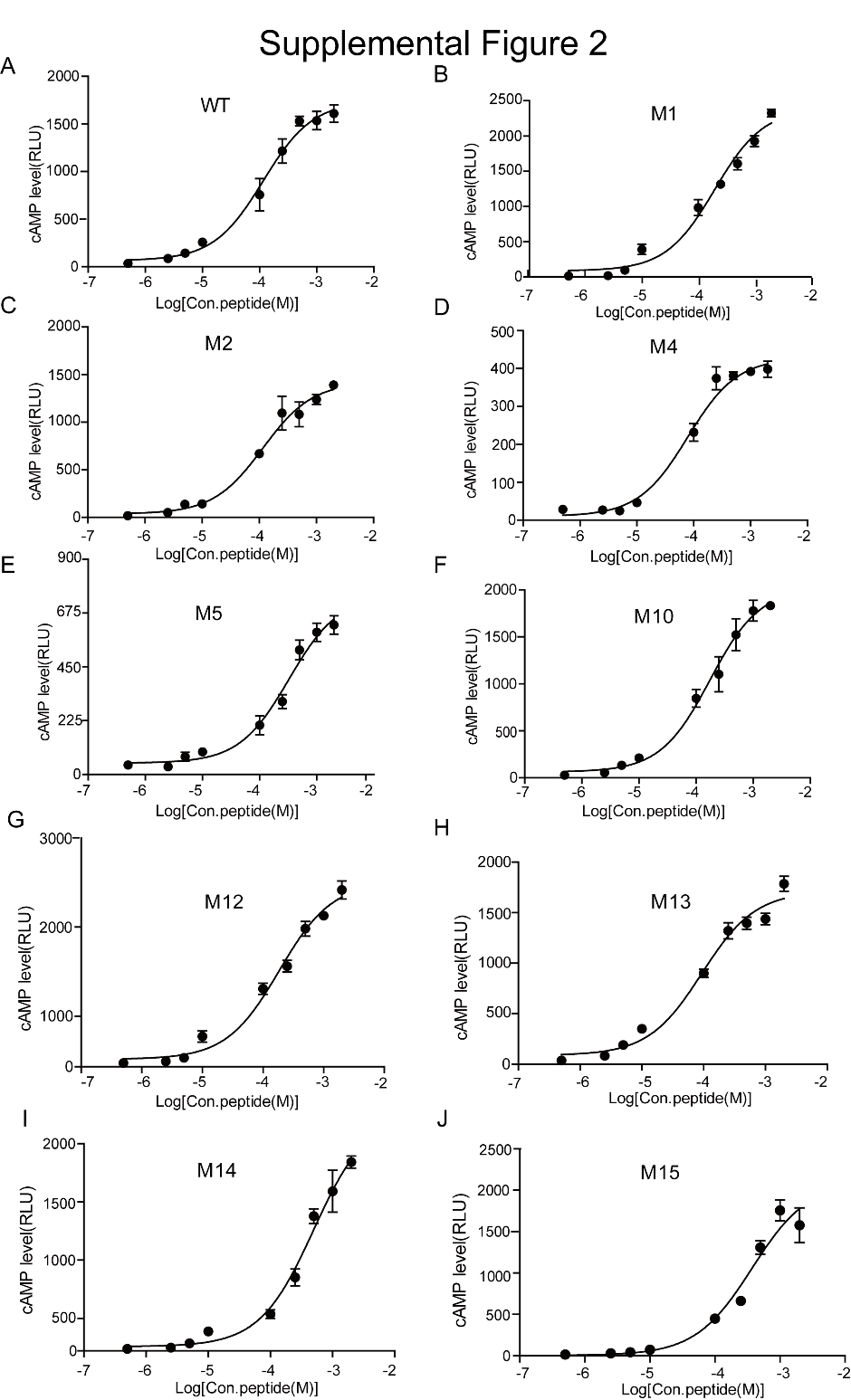


**Supplemental Figure 2. Signaling properties of ADGRG2 activated by p15 (WT) and its different mutants.**

**A-J.** Signaling properties of ADGRG2-△GPS-β activated by p15(WT, A) and its different mutants (p15-Muts B-J). HEK293 cells transfected with ADGRG2-FL were stimulated by increasing the concentration of ADGRG2 *Stachel* peptide p15. cAMP levels were detected by the Glosensor assay. Each experiment was repeated for 6 times.


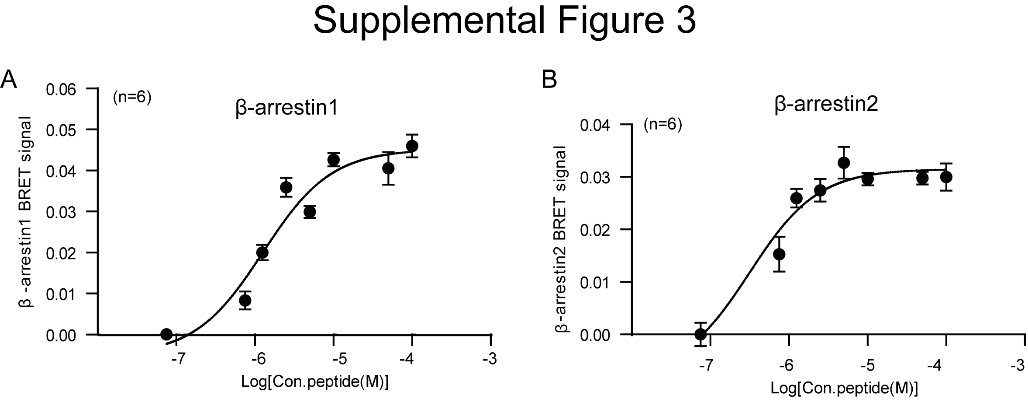


**Supplemental Figure 3.** **β-arrestin1 or β-arrestin2 recruitment signaling properties of ADGRG2-β (ADGRG2-GPS-truncation) activated by ADGRG2 *Stachel* peptide p15 T1V/F3Phe(4-Me).**

**A-B.** β-arrestin1 or β-arrestin2 recruitment stimulated by increasing the concentration of ADGRG2 *Stachel* peptide p15 T1V/F3Phe(4-Me) of ADGRG2-β (ADGRG2-GPS-truncation) were detected by BRET.


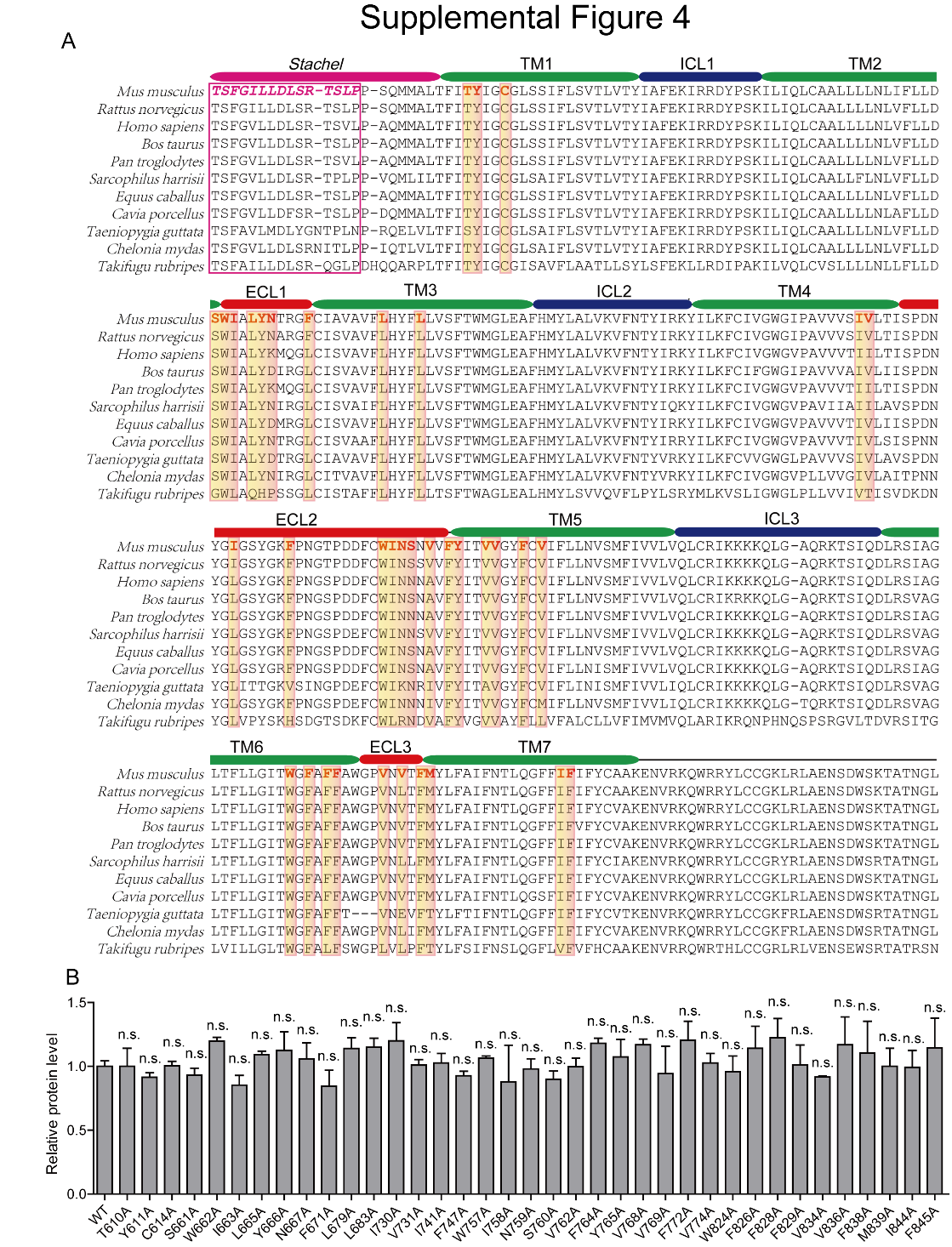


**Supplemental Figure 4. Designed ADGRG2 mutants to characterize the ADGRG2** ***Stachel* peptide binding mode.**

**A.** Sequence alignment of the transmembrane domains of ADGRG2 (*Mus musculus, Rattus norvegicus, Homo sapiens, Bos taurus, Pan troglodytes, Sarcophilus harrisii, Equus caballus, Cavia porcellus, Taeniopygia guttata, Chelonia mydas, Takifugu rubripes*). The designed mutations are highlighted. **B.** Relative expression levels of ADGRG2-△GPS-β WT and mutants. HEK293 cells were transfected with ADGRG2-△GPS-β WT and mutants, expression levels were determined by cell-surface ELISA and were adjusted for the amount of transfected plasmid to assure equal expression. Data are normalized by expression levels to the ADGRG2-△GPS-β WT. n.s., no significant differences. Relative receptor expression levels of ADGRG2-△GPS-β mutants were compared with ADGRG2-△GPS-β WT. Each experiment was repeated for 3 times.


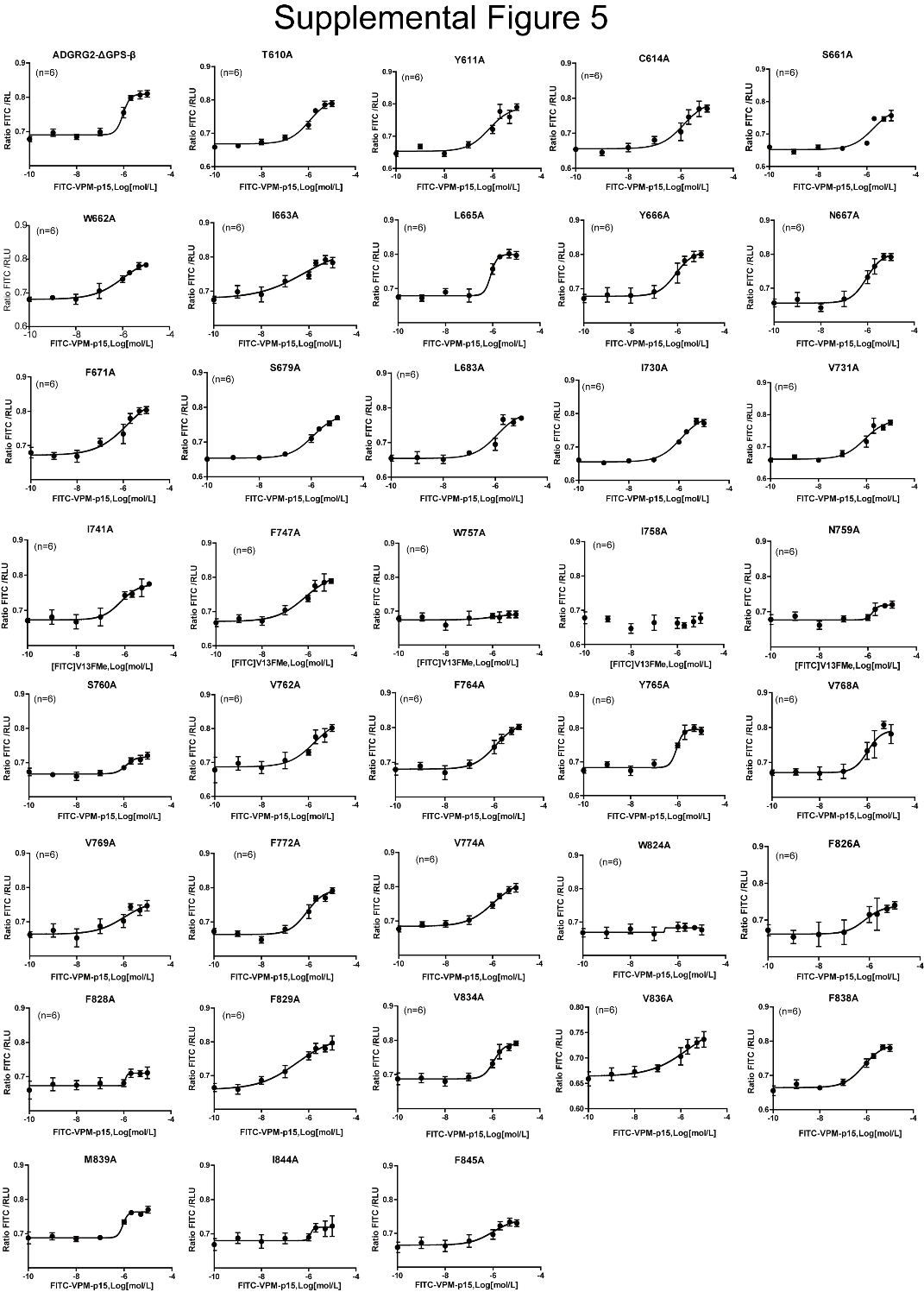


**Supplemental Figure 5.** Binding curves of ADGRG2-△GPS-β WT or its mutants toward VPM-p15 (p15-T1V/F3Phe(4-Me)) were determined using the BRET assay and FITC-conjugated VPM-p15 peptide. Each experiment was repeated for 6 times.


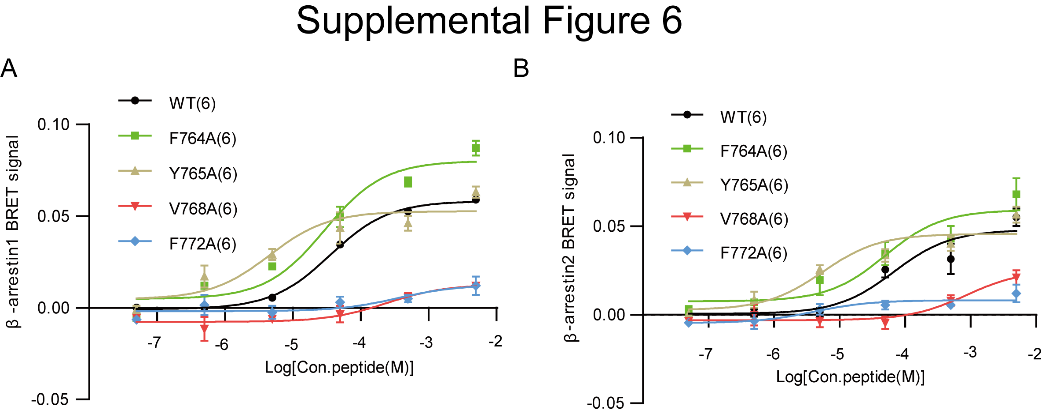


**Supplemental Figure 6. Detection of β-arrestin1 or β-arrestin2 recruitment by mutations affecting G protein signal transduction.**

**A-B.** β-arrestin1(A) or β-arrestin2(B) recruitment signaling were detected by the BRET assay. Signaling profiles of the truncated ADGRG2 β subunit and its mutants that affect G protein signaling in response to the ADGRG2 *Stachel* peptide VPM-p15 derived from ADGRG2 *Stachel* sequence. HEK293 cells were transfected with ADGRG2-△GPS-β or its mutants, cells transfected with pcDNA3.1 were used as control. Each experiment was repeated for 6 times.

**Supplemental Table 1. Bmax and EC_50_ of HEK293 cells transfected with ADGRG2-FL were stimulated by increasing concentration of the p15 (WT) and its different mutants**

| Mutants | Bmax | EC_50_ |
| --- | --- | --- |
| WT | 1832 | 1.752±0.523e-004 |
| T1A | 2322 | 1.341±0.284e-004 |
| S2A | 1516 | 2.100±0.355e-004 |
| G4A | 504 | 3.100±0.504e-004 |
| I5A | 751 | 3.260±0.694e-004 |
| S10A | 2018 | 1.753±0.395e-004 |
| T12A | 2347 | 1.290±0.347e-004 |
| S13A | 1831 | 1.722±0.456e-004 |
| L14A | 1938 | 2.738±0.583e-004 |
| P15A | 1993 | 1.622±0.598e-004 |

**Supplemental Table 2. Bmax and EC_50_ of the optimization** **ADGRG2 *Stachel* peptide p15**

|  | WT | T1V | F3Phe(4-Me) | F3 1-Nal | T1V/F3 1-Nal | T1V/F3Phe(4-Me) |
| --- | --- | --- | --- | --- | --- | --- |
| Bmax | 6531 | 6683 | 6481 | 6280 | 6608 | 6654 |
| EC_50_(mol/L) | 2.43±0.33  e-004 | 7.68±1.65  e-005 | 9.86±1.98  e-005 | 5.55±1.55  e-005 | 4.70±1.21  e-006 | 1.41±0.16  e-006 |

**Supplemental Table 3. Statistical Table of Binding of Different Mutants of ADGRG2 to FITC-VPM-p15**

| Mutants name | EC_50_(mol/L) | Fmut | Bmax |
| --- | --- | --- | --- |
| WT | 9.358±0.119e-007 | 1.000 | 0.809±0.015 |
| T610A | 1.120±0.139 e-006 | 1.197 | 0.8072±0.024 |
| Y611A | 7.124±0.138 e-007 | 0.761 | 0.7912±0.020 |
| C614A | 1.294±0.137 e-006 | 1.382 | 0.7934±0.0241 |
| S661A | 7.784±0.147 e-007 | 0.832 | 0.6895±0.021 |
| W662A | 1.056±0.043e-006 | 1.128 | 0.808±0.047 |
| I663A | 7.721±0.146e-007 | 0.825 | 0.814±0.043 |
| L665A | 7.955±0.120e-007 | 0.850 | 0.799±0.012 |
| Y666A | 8.378±0.131e-007 | 0.895 | 0.809±0.029 |
| N667A | 9.128±0.147e-007 | 0.975 | 0.803±0.028 |
| F671A | 8.799±0.179e-007 | 0.940 | 0.833±0.050 |
| L679A | 1.162±0.127e-006 | 1.242 | 0.7804±0.017 |
| L683A | 1.160±0.134 e-006 | 1.240 | 0.7887±0.022 |
| I730A | 1.199±0.140e-006 | 1.281 | 0.7950±0.031 |
| V731A | 8.382±0.124 e-007 | 0.944 | 0.7903±0.018 |
| I741A | 6.694±0.085e-007 | 0.715 | 0.775±0.093 |
| F747A | 7.590±0.142e-007 | 0.811 | 0.813±0.041 |
| W757A |  | No binding |  |
| I758A |  | No binding |  |
| N759A | 1.549±0.042e-006 | 1.655 | 0.713±0.011 |
| S760A | 1.347±0.049e-006 | 1.444 | 0.716±0.011 |
| V762A | 1.187±0.142e-006 | 1.268 | 0.798±0.021 |
| F764A | 1.107±0.128e-006 | 1.183 | 0.814±0.039 |
| Y765A | 9.052±0.113e-007 | 0.967 | 0.796±0.013 |
| V768A | 1.041±0.109e-006 | 1.112 | 0.792±0.029 |
| V769A | 1.072±0.098e-006 | 1.145 | 0.763±0.074 |
| F772A | 8.425±0.132e-007 | 0.903 | 0.795±0.021 |
| V774A | 1.075±0.136e-006 | 1.149 | 0.820±0.032 |
| W824A |  | No binding |  |
| F826A | 1.168±0.011e-006 | 1.248 | 0.734±0.035 |
| F828A | 1.166±0.036e-006 | 1.247 | 0.706±0.006 |
| F829A | 3.226±0.158e-007 | 0.344 | 0.817±0.050 |
| V834A | 1.120±0.102e-006 | 1.196 | 0.789±0.016 |
| V836A | 1.230±0.050e-006 | 1.315 | 0.775±0.030 |
| F838A | 7.666±0.128e-007 | 0.819 | 0.793±0.022 |
| M839A | 8.956±0.076e-007 | 0.957 | 0.764±0.008 |
| I844A | 1.535±0.039e-006 | 1.640 | 0.729±0.093 |
| F845A | 1.358±0.07e-006 | 1.452 | 0.749±0.071 |

The binding parameters. The mutation factor (Fmut) is calculated as K*d* (mutated ADGRG2-ΔGPS-β)/K*d*(WT-ADGRG2-ΔGPS-β).

**Supplemental Table 4. Bmax and EC_50_ of the optimization ADGRG2 agonistic peptide p15 to different ADGRG2 mutants**

| Mutants name | EC_50_(mol/L) | Bmax | Mutants name | EC_50_(mol/L) | Bmax |
| --- | --- | --- | --- | --- | --- |
| WT | 1.06±0.124e-006 | 6034 | S760A | 1.954±0.109 e-006 | 1884 |
| T610A | 1.032±0.038e-006 | 4023 | V762A | 2.345±0.075e-006 | 4386 |
| Y611A | 1.065±0.132e-006 | 3894 | F764A | 0.938±0.075e-006 | 6345 |
| C614A | 2.169±0.276e-006 | 3408 | Y765A | 1.067±0.048 e-006 | 6413 |
| S661A | 2.319±0.234e-006 | 2830 | V768A | 7.392±0.274e-006 | 779 |
| W662A | 1.548±0.069e-006 | 2110 | V769A | 1.691±0.098e-006 | 2431 |
| I663A | 1.201±0.078e-006 | 5079 | F772A | 2.185±0.254e-006 | 1173 |
| L665A | 1.593±0.082e-006 | 4196 | V774A | 1.281±0.148e-006 | 5605 |
| Y666A | 1.127±0.118e-006 | 2616 | W824A | 9.754±0.132e-006 | 123.6 |
| N667A | 1.241±0.017e-006 | 2698 | F826A | 6.600±0.083e-006 | 1422 |
| F671A | 1.202±0.110e-006 | 3916 | F828A | 3.880±0.083e-006 | 1572 |
| L679A | 1.162±0.114e-006 | 5023 | F829A | 1.158±0.077e-006 | 8572 |
| L683A | 1.314±0.107e-006 | 5488 | V834A | 1.137±0.056e-006 | 1912 |
| I730A | 1.332±0.106e-006 | 5716 | V836A | 4.127±0.616e-006 | 1258 |
| V731A | 1.223±0.105e-006 | 5619 | F838A | 1.700±0.058e-006 | 2887 |
| I741A | 1.554±0.105e-006 | 3435 | M839A | 1.721±0.153e-006 | 2681 |
| F747A | 1.130±0.125e-006 | 5631 | I844A | 12.92±1.863e-006 | 343.8 |
| W757A | 14.23±0.413e-006 | 114.3 | F845A | 3.209±0.807e-006 | 1460 |
| I758A | 13.81±0.670e-006 | 107.4 |  |  |  |
| N759A | 4.758±0.263e-006 | 2507 |  |  |  |

**Supplemental Table 5. Primer sequences for ADGRG2-ΔGPS-β mutants**

| Primer name | Primer sequence 5’-3’ | |
| --- | --- | --- |
| T610A-F | CGCATATATTGGCTGTGGGCTTTCATC | |
| T610A-R | CACAGCCAATATATGCGATAAATGTCAGAGCCATCATTTGA | |
| Y611A-F | TATCACGGCAATTGGCTGTGGGCTTTCATC | |
| Y611A-R | AGCCAATTGCCGTGATAAATGTCAGAGCCATCA | |
| C614A-F | TATTGGCGCAGGGCTTTCATCAATTTTTCTGTC | |
| C614A-R | AAAGCCCTGCGCCAATATACGTGATAAATGTCAGAG | |
| S661A-F | CCTAGACGCATGGATTGCGCTGTATAATACCC | |
| S661A-R | CAATCCATGCGTCTAGGAGGAAGATCAGGTTGAG | |
| W662A- F | GATCTTCCTCCTAGACTCCGCGATTGCGCTGTATAATACC | |
| W662A- R | GGTATTATACAGCGCAATCGCGGAGTCTAGGAGGAAGATC | |
| I663A- F | CTCCTAGACTCCTGGGCTGCGCTGTATAATAC | |
| I663A- R | GTATTATACAGCGCAGCCCAGGAGTCTAGGAG | |
| L665A- F | CTCCTGGATTGCGGCGTATAATACCCGAG | |
| L665A- R | CTCGGGTATTATACGCCGCAATCCAGGAG | |
| Y666A- F | CTGGATTGCGCTGGCTAATACCCGAGG | |
| Y666A- R | CCTCGGGTATTAGCCAGCGCAATCCAG | |
| N667A- F | GGATTGCGCTGTATGCTACCCGAGGTTTCTG | |
| N667A- R | CAGAAACCTCGGGTAGCATACAGCGCAATCC | |
| F671A- F | GTATAATACCCGAGGTGCCTGCATTGCCGTGGC | |
| F671A- R | GCCACGGCAATGCAGGCACCTCGGGTATTATAC | |
| L679A-F | TGTATTTGCAACCCCTCCAAAATCCTCATCC | |
| L679A-R | GAGGGGTTGCAAATACAGCCACGGCAATGC | |
| L683A-F | GCATTGGTCTCATTCACATGGATGGGATTAGA | |
| L683A-R | GTGAATGAGACCAATGCAAAATAGTGAAGAAATACAGCCACGG | |
| I730A-F | TCCGCAGTCCTGACTATATCCCCAGATAACTATG | |
| I730A-R | ATAGTCAGGACTGCGGACACAACCACAAAATACAGCC | |
| V731A-F | GTCCATCGCACTGACTATATCCCCAGATAACTATGG | |
| V731A-R | TAGTCATGCGATGGACACAACCACAAAATACAG | |
| I741A- F | GATAACTATGGGGCTGGATCCTATGG | |
| I741A- R | CCATAGGATCCAGCCCCATAGTTATC | |
| F747A- F | GGATCCTATGGAAAAGCCCCCAATGGCACACC | |
| F747A- R | GGTGTGCCATTGGGGGCTTTTCCATAGGATCC | |
| W757A- F | CAGATGACTTTTGCGCGATCAACAGCAATG | |
| W757A-R | CATTGCTGTTGATCGCGCAAAAGTCATCTG | |
| I758A- F | GATGACTTTTGCTGGGCCAACAGCAATGTGGTG | |
| I758A- R | CACCACATTGCTGTTGGCCCAGCAAAAGTCATC | |
| N759A- F | GACTTTTGCTGGATCGCCAGCAATGTGGTGTTC | |
| N759A- R | GAACACCACATTGCTGGCGATCCAGCAAAAGTC | |
| S760A- F | GACTTTTGCTGGATCAACGCCAATGTGGTGTTCTATATC | |
| S760A- R | GATATAGAACACCACATTGGCGTTGATCCAGCAAAAGTC | |
| V762A- F | GATCAACAGCAATGCGGTGTTCTATATC | |
| V762A- R | GATATAGAACACCGCATTGCTGTTGATC | |
| F764A- F | CAACAGCAATGTGGTGGCCTATATCACGGTTGTG | |
| F764A- R | CACAACCGTGATATAGGCCACCACATTGCTGTTG | |
| Y765A- F | CAATGTGGTGTTCGCTATCACGGTTGTGG | |
| Y765A- R | CCACAACCGTGATAGCGAACACCACATTG | |
| V768A- F | GTTCTATATCACGGCTGTGGGATATTTCTG | |
| V768A- R | CAGAAATATCCCACAGCCGTGATATAGAAC | |
| V769A- F | CTATATCACGGTTGCAGGATATTTCTGTGTG |  |
| V769A- R | CACACAGAAATATCCTGCAACCGTGATATAG |  |
| F772A-F | CACGGTTGTGGGATATGCCTGTGTGATATTTCTAC |  |
| F772A-R | GTAGAAATATCACACAGGCATATCCCACAACCGTG |  |
| V774A-F | GTGGGATATTTCTGTGCGATATTTCTACTGAACG |  |
| V774A-R | GTGGGATATTTCTGTGCGATATTTCTACTGAACG |  |
| W824A- F | CTGGGAATTACTGCGGGCTTTGCCTTC |  |
| W824A- R | GAAGGCAAAGCCCGCAGTAATTCCCAG |  |
| F826A- F | GGAATTACTTGGGGCGCTGCCTTCTTTGCCTG |  |
| F826A- R | CAGGCAAAGAAGGCAGCGCCCCAAGTAATTCC |  |
| F828A- F | GAATTACTTGGGGCTTTGCCGCCTTTGCCTGGGGACCAG | |
| F828A- R | CTGGTCCCCAGGCAAAGGCGGCAAAGCCCCAAGTAATTC | |
| F829A- F | GGCTTTGCCTTCGCTGCCTGGGGACCAGTTAATG | |
| F829A- R | CATTAACTGGTCCCCAGGCAGCGAAGGCAAAGCC | |
| V834A- F | GCCTGGGGACCAGCTAATGTCACCTTC | |
| V834A- R | GAAGGTGACATTAGCTGGTCCCCAGGC | |
| V836A- F | GGACCAGTTAATGCCACCTTCATGTATC | |
| V836A- R | GATACATGAAGGTGGCATTAACTGGTCC | |
| F838A- F | CCAGTTAATGTCACCGCGATGTATCTCTTTGCC | |
| F838A- R | GGCAAAGAGATACATCGCGGTGACATTAACTGG | |
| M839A- F | GTTAATGTCACCTTCGCGTATCTCTTTGCCATC |  |
| M839A- R | GATGGCAAAGAGATACGCGAAGGTGACATTAAC |  |
| I844A- F | CATGTATCTCTTTGCCGCCTTTAACACCTTACAAG |  |
| I844A- R | CTTGTAAGGTGTTAAAGGCGGCAAAGAGATACATG |  |
| F845A- F | GTATCTCTTTGCCATCGCTAACACCTTACAAGG |  |
| F845A- R | CCTTGTAAGGTGTTAGCGATGGCAAAGAGATAC |  |
